# US regulatory approaches to chemistry, manufacturing, and controls for botanical drug products

**DOI:** 10.1186/1479-5876-10-S2-A40

**Published:** 2012-10-17

**Authors:** Rajiv Agarwal

**Affiliations:** 1 Office of New Drug Quality Assessment, Office of Pharmaceutical Science, US Food and Drug Administration, 10903 New Hampshire AvenueSilver Spring, MD 20903, USA

## 

Many botanical products are used widely in the United States. Depending on its labeling and intended use, a botanical product can be a food, a dietary supplement, and/or a drug. If a botanical product is intended for use in diagnosing, mitigating, treating, or curing disease, it is a drug under the Food, Drug, and Cosmetic Act and is subject to applicable drug regulations. The CDER Guidance on Botanical Drug Products defines the term “Botanical” as a finished, labeled product that contains drug substance from plant origins, which may include plants or plant parts, algae, macroscopic fungi, and combinations thereof. The term does not include highly purified substances or chemically modified substances derived from botanical sources. This presentation provides an overview of the Chemistry, Manufacturing, and Controls (CMC) information recommended to support the clinical studies of a botanical drug product under an investigational new drug application (IND) in the United States. Veregen (sinecatachins) ointment1 was the first botanical drug product approved by the FDA since publication of the Botanical Guidance (2004)2. While the Guidance gave a general pathway for IND submissions, it was silent on the requirements for NDA approval. A "CDER Botanical Guidance rewrite Working Group" has been established and the work is in progress to consolidate the FDA's current scientific and regulatory thinking in the future Botanical Guidance.
